# Comments on the recent changes in taxonomy of pygmy unicorns, with description of a new species of *Metopomystrum* from Brazil (Insecta, Tetrigidae, Cleostratini, Miriatrini)

**DOI:** 10.3897/zookeys.702.13981

**Published:** 2017-09-25

**Authors:** Daniela Santos Martins Silva, Josip Skejo, Marcelo Ribeiro Pereira, Fernando Campos De Domenico, Carlos Frankl Sperber

**Affiliations:** 1 SIGTET-Special Interest Group Tetrigidae; 2 Programa de Pós-Graduação em Entomologia, Departamento de Entomologia, Universidade Federal de Viçosa (UFV), Avenida PH Rolfs, s/n, CEP: 36570-900, Viçosa – MG, Brazil; 3 University of Zagreb, Faculty of Science, Department of Biology, Rooseveltov trg 6, HR-10000 Zagreb, Croatia; 4 Instituto de Ciências Biológicas e da Saúde, Universidade Federal de Viçosa (UFV) – campus Rio Paranaíba, Rodovia MG 230, KM 7, s/n, CEP: 38810-000, Rio Paranaíba – MG, Brazil; 5 Museu de Zoologia da Universidade de São Paulo, Universidade de São Paulo (USP), Avenida Nazaré, 481, CEP: 04263-000, São Paulo – SP, Brazil; 6 Departamento de Biologia Geral, Universidade Federal de Viçosa (UFV), Avenida PH Rolfs, s/n, CEP: 36570-900, Viçosa – MG, Brazil

**Keywords:** *Apteromystrum*, Atlantic Forest, fastigium, pygmy grasshopper, taxonomy

## Abstract

The tribe Cleostratini Bolívar, 1887 *sensu* Storozhenko, 2016 does not represent a monophyletic taxon because it gathers various Tetrigidae genera with various types of horn and prolongation of frons or vertex. Prolongation of these structures is present in morphologically and biogeographically distant groups. We do not regard Miriatrini Cadena-Castañeda & Cardona, 2015 synonymous with Cleostratini because the genus *Miriatra* Bolívar, 1906 belongs to a group of genera distant from *Cleostratus* Stål, 1877. There is no adequate diagnosis for proposed groups of genera forming tribes Cleostratini or Miriatrini. Miriatrini **stat. resurr.** are monotypic and include only *Miriatra*, Cleostratini are monotypic as well. *Apteromystrum* Storozhenko, 2016 **syn. n.** is regarded synonymous with *Metopomystrum*, *M. apterum* **comb. resurr.**, *M. amazoniensis* **comb. resurr.** and *Miriatra brevifastigiata* (Cadena-Castañeda & Cardona, 2015), **comb. n. **are not *Metopomystrum* member. Herein a new species of pygmy unicorn, *Metopomystrum muriciense* Silva & Skejo, **sp. n.**, is described from Atlantic Forest remnants in northeast of Brazil, collected on the Estação Serra do Ouro (municipality of Murici, Alagoas state). Distribution data, morphological characterization, and an identification key to *Metopomystrum* species are also presented.

## Introduction

Members of the family Tetrigidae are distributed all over the world (Hancock 1907), in almost all climatic zones except deserts and regional New Zealand (Rehn 1952, Tumbrinck 2014). Pygmy grasshoppers usually live close to water, decomposing soil and leaf litter (Hancock 1902). They are more active in warm temperatures and are extremely difficult to find because they move very slowly in the substrate (Ragge 1965). Currently, the family includes about 1900 species within about 280 genera within eight subfamilies Batrachideinae Bolívar, 1887, Cladonotinae Bolívar, 1887, Discotettiginae Hancock, 1907, Lophotettiginae Hancock, 1909, Metrodorinae Bolívar, 1887, Scelimeninae Bolívar, 1887, Tetriginae Rambur, 1838 and Tripetalocerinae Bolívar, 1887 (Cigliano et al. 2017).

The subfamily Metrodorinae is not a monophyletic, but polyphyletic group established for practical identification (Pavón-Gozalo et al. 2012). The subfamily was erected by Bolivar in 1887 with the following diagnostic characters: lateral lobes of the pronotum directed sidewards (in rare cases downwards, similarly to Tetriginae), obliquely truncated behind and very rarely bearing acute spines (similar to Scelimeninae). In 2015 Cadena-Castañeda and Cardona established the tribe Miriatrini to gather all the genera with prolonged fastigium of the vertex within the subfamily Metrodorinae. The tribe included two Neotropical genera *Metopomystrum* Günther, 1939 (five species) and *Miriatra* Bolívar, 1906 (four species), and nine more genera from other biogeographic regions, namely *Corystotettix* Günther, 1939 (Indonesia, one species); *Indomiriatra* Tinkham, 1939 (India, one species); *Procytettix* Bolívar, 1912 (Indian Ocean Islands, four species); *Rhynchotettix* Hancock, 1907 (Madagascar, two species); *Rhopalotettix* Hancock, 1910 (Asia and China, nine species); *Rostella* Hancock, 1913 (Asia, two species); *Spadotettix* Hancock, 1910 (India, Sri Lanka, China and Asia, five species); *Thibron* Rehn, 1939 (Africa, five species) and *Thyrsus* Bolívar, 1887 (Fiji and New Guinea, two species). The following characters are included in the Miriatrini diagnosis: long face; elevated vertex, exceeding length of scape and pedicel; ovoid eyes (in lateral view); narrow frontal costa with base connected to fastigium; pronotum dorsally flattened and elongated, as long as alae, lateral lobes of pronotum not expanded or with lateral spines (Cadena-Castañeda and Cardona 2015).

The subfamily Cleostratinae was established by Bolívar in 1887 as ‘sectio Cleostratae’ for a single genus *Cleostratus* Stål, 1877, unique among Tetrigidae in having antennal grooves and the median ocellus placed between the compound eyes and an extremely produced frons. Recently, Storozhenko (2016) transformed the subfamily Cleostratinae into the tribe Cleostratini and placed it within Metrodorinae subfamily. Storozhenko (2016) regards Miriatrini synonymous with Cleostratini. The tribe Cleostratini *sensu* Storozhenko now gathers the following genera: (1) *Apteromystrum* Storozhenko, 2016 (established for wingless *Metopomystrum*), (2) *Cleostratus*, (3) *Halmahera* Storozhenko, 2016, (4) *Indomiriatra*, (5) *Metopomystrum*, (6) *Miriatra*, (7) *Miriatroides* Zheng & Jiang, 2002, (8) *Procytettix*, (9) *Pseudomitraria* Hancock, 1907, (10) *Rhopalina* Tinkham (= *Corystotettix*), 1939, (11) *Rhopalotettix*, (12) *Rhynchotettix*, (13) *Rostella*, (14) *Spadotettix*, (15) *Thyrsus*, and (16) *Uvarovithyrsus* Storozhenko, 2016 (established for *Thyrsus* from New Guinea). All the genera are grouped together on the basis of a single character: presence of a horn-like structure on the head (frons or fastigium of vertex well developed and projected into long or relatively short protuberance directed forwards or upwards).

The genus of pygmy unicorns *Metopomystrum* Günther, 1939 (Günther 1939, pp. 270) is a South American genus that includes five species if old taxonomy is followed (e.g., Günther 1939; Cadena-Castañeda and Cardona 2015), namely (1) *M. amazoniensis* Cadena-Castañeda and Cardona, 2015; (2) *M. brevifastigiata* Cadena-Castañeda and Cardona, 2015; (3) *M. lilianae* Cadena-Castañeda and Cardona, 2015; (4) *M. pehlkei* Günther, 1939 and (5) *M. apterum* Günther, 1939. Storozhenko (2016) divided the genus into two genera, one consisting of winged species: *Metopomystrum* including *M. pehlkei* and *M. lilianae* and the other of wingless species: *Apteromystrum* including *A. amazoniensis* (Cadena-Castañeda and Cardona, 2015), *A. brevifastigiata* (Cadena-Castañeda and Cardona, 2015) and *A. apterum* (Günther, 1939).

Our aims are (1) to describe a new species of pygmy unicorn from Atlantic Forest in the state of Alagoas, Brazil, by providing distribution data, morphological characterization and an identification key for species of the genus, (2) to test if there are relevant differences between *Metopomystrum* and *Apteromystrum* regardless of the presence of wings and (3) to discuss taxonomic and evolutionary aspects of Cleostratinae, Cleostratini, and Miriatrini.

## Materials and methods

**Sampling and study area.** The specimen of the new species of pygmy unicorn was collected by the “Biota de Orthoptera do Brasil” research group, 21–23 January 2013 at the Estação Serra do Ouro, municipality of Murici (state of Alagoas, Brazil) (coordinates 9°14.54'S, 35°50.2'W), with a pitfall trap containing ethanol fuel killing solution.

**Microscopy and photography.** External morphological characteristics were examined using a Zeiss Stemi 2000 stereomicroscope and photographed with Zeiss Stereo Discovery V20 stereomicroscope. Photographs were taken with the multidimensional acquisition function with AXIO VISION software, which allows capturing a series of pictures at different focal planes. The resulting images were then combined into a single picture using the Extended Focus Z function. The image plates were prepared in image editing software. Photographs of lateral and dorsal view of the holotype were taken, as well as more detailed photographs of important morphological characters (antennal segments, frontal costa, fastigium, vertex, maxillary palp, lateral lobes of the pronotum, sternomentum, abdomen (lateral and ventral profile), forelegs, hind femur, supranal plate, cerci, and subgenital plate.

**Terminology.** Morphological terminology follows Devriese (1996) and Tumbrinck (2014). Measurements and character description are based on Tumbrinck (2014).

**Figure 1.**
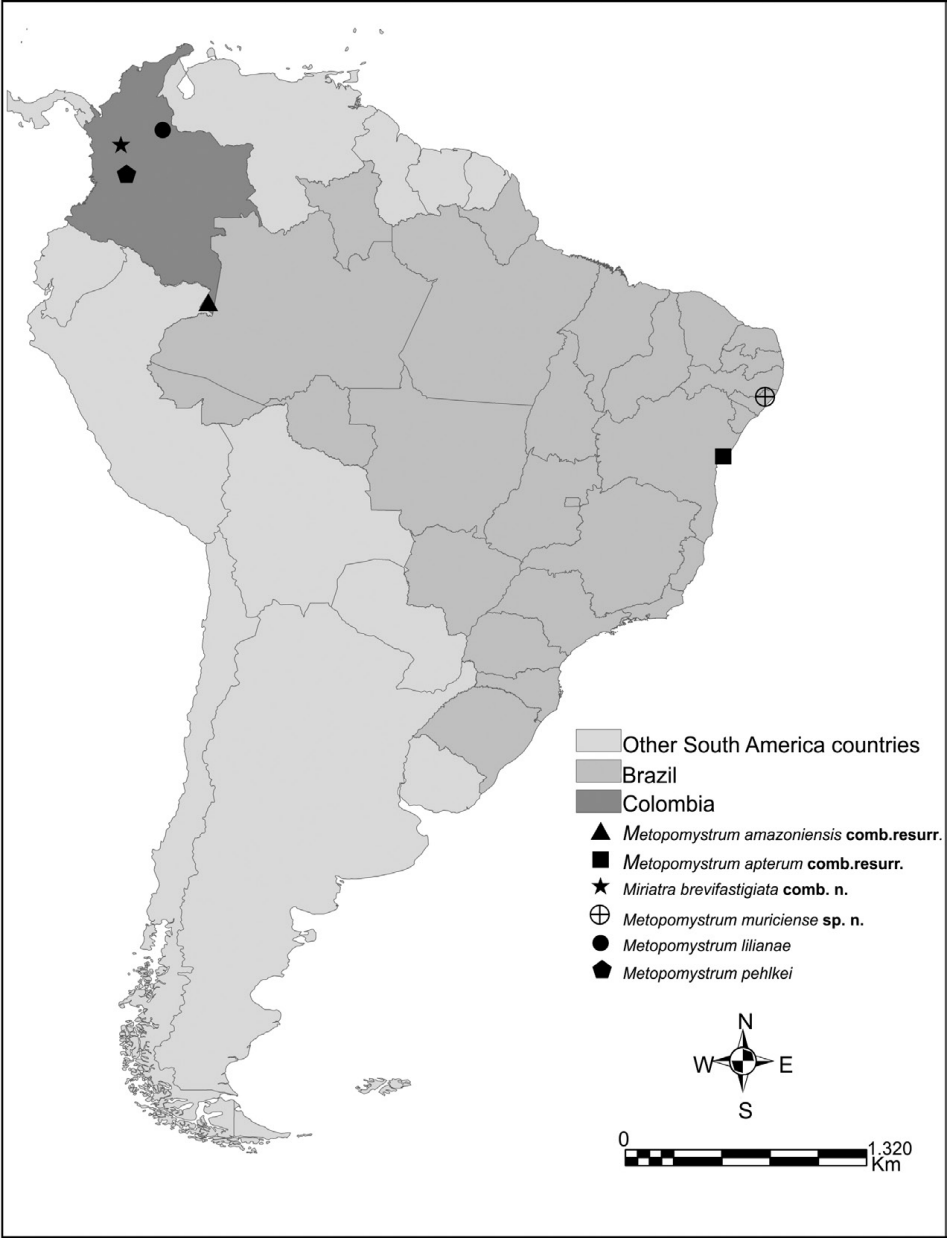
Distribution map of *Metopomystrum* with *Miriatra brevifastigiata* comb. n., before Storozhenko (2016).

**Distribution map and depository of type specimen.** Distribution map (Fig. 1) was produced with all available distribution records of the species of *Metopomystrum* (before Storozhenko, 2016). Geographical coordinates of localities were estimated using the information available in the literature (Table 1). The holotype of the new species is deposited in the Museu Nacional da Universidade Federal do Rio de Janeiro (**MNRJ**).

**Table 1.** Distribution data for hitherto described species of the genus *Metopomystrum*.

Species	Type series (holotype sex, locality, depository)	References
*Metopomystrum amazoniensis* comb. resurr.	1♀, Colombia: Amazonas, PNN Amacayacu (Universidad Distrital Francisco José de Caldas, Colección de Entomología y Aracnología, Bogota, Colombia)	Cadena-Castañeda and Cardona (2015)
*Metopomystrum apterum* comb. resurr.	1♂, Brazil Northeast: Bahia, Freire* (Museum für Naturkunde, Berlin, Germany)	Günther (1939)
*Miriatra brevifastigiata* comb. n.	1♂ (nymph), Colombia: Antioquia, Envigado (Universidad Distrital Francisco José de Caldas, Colección de Entomología y Aracnología, Bogota, Colombia)	Cadena-Castañeda and Cardona (2015)
*Metopomystrum lilianae*	1♂, Colombia, Colombia: Santander, Puerto Parra, Campo Capote, Borojó (Universidad Distrital Francisco José de Caldas, Colección de Entomología y Aracnología, Bogotá, Colombia)	
*Metopomystrum pehlkei*	1♂ Colombia: Tolima, Hacienda Pehlke, Städtisches Museum Stettin, Szczecin, Poland	

* We could not find any locality with this name in the state of Bahia (Brazil).

## Results

### Taxonomic comment on *Metopomystrum brevifastigiata*

The species *Metopomystrum brevifastigiata* Cadena-Castañeda and Cardona, 2015 is not member of *Metopomystrum* nor *Apteromystrum*, but its holotype represents a male nymph of *Miriatra* sp. The holotype of *M. brevifastigiata* lacks antegenicular teeth on the hind femora which implies that it is not an adult animal. Head and pronotal morphology is also rather different from *Metopomystrum* and *Apteromystrum* species and represents a typical set of characters defining *Miriatra*. Thus, the new combination *Miriatra brevifastigiata* (Cadena-Castañeda and Cardona, 2015) comb. n. should be used when referring to this specimen. This species is very close to *Miriatra producta* (Bolívar, 1887) and the differences should be checked when adults will be found.

### Re-evaluation of diagnostic characters separating *Metopomystrum* and *Apteromystrum*

Storozhenko (2016) established the genus *Apteromystrum* and separated it from *Metopomystrum* on the basis of lack of the flying organs. Diagnosis is not exactly stated in the descriptive paper, but should be derived from the key. Thus, here we briefly compare main features of *Metopomystrum* and *Apteromystrum*, including characters of a new species that should be placed within *Apteromystrum* after Storozhenko’s (2016) division into a winged (*Metopomystrum*) and an apterous group (*Apteromystrum*). From Storozhenko (2016) the diagnostic features of those genera are (after combination of characters from the key): ***Metopomystrum*** (after Storozhenko 2016, every character is shortened with M and number for discussion): (M1) antennal grooves situated in the level of the lower margin of the compound eyes or slightly above it; (M2) frontal ridge in lateral view with weak excision between the compound eyes; (M3) median ocellus situated distinctly below the lower margin of the compound eyes; (M4) fastigium of the vertex long and produced forwards with pointed, triangular or broadly rounded apex, its length equal or greater than the length of an eye; (M5) median carina of the vertex absent, or if present weak; (M6, corresponds to A1) ventral and tegminal sinuses on the paranota well developed, tegmina and wings visible, wings reaching the apex of the posterior process of the pronotum; (M7) lower side of lateral lobes of the pronotum in dorsal view without spine; (M8) fore and mid femora without lobes on upper margin and (M9) ventral margins of femora straight or slightly undulated. *Apteromystrum* (after Storozhenko, every character is shortened with A and number for discussion): (A1, corresponds to M6) paranota only with ventral sinus, lacking tegminal sinus, tegmina and wings absent or not visible; (A2) fastigium of the vertex with continuous lateral margins; (A3, corresponds to M5) fastigium lacking median carina.

It is evident that comparison of *Metopomystrum* and the new genus *Apteromystrum* in Storozhenko (2016) is not completely satisfactory. For most of the diagnostic characters of *Metopomystrum* (M1, M2, M3, M4, M7, M8, M9) there is no comparison with corresponding characters of *Apteromystrum* and there are only three *Apteromystrum* characters, which is not satisfactory as generic diagnosis, because the main diagnostic character for *Apteromystrum* is the lack of visible flight organs (A1 contrary to M6), while A3 is the same as M5 and for A2 there is no comparison with *Metopomystrum*. Concerning diagnostic characters, there are no significant differences in morphology, except for lack of wings in *Apteromystrum*. However, apterous *A. amazoniensis* morphologically looks more similar to biogeographically closer *M. pehlkei* and *M. lilianae* than to *A. muriciense* Silva & Skejo, sp. n. and *A. apterum* from Brazil. Thus, we do not regard the division in two genera necessary but regard *Apteromystrum* Storozhenko, 2016, syn. n. synonymous with *Metopomystrum*. Here we provide a redescription and diagnosis of the genus.

#### Genus Metopomystrum

Taxon classificationAnimaliaOrthopteraTetrigidae

Günther, 1939

= Apteromystrum Storozhenko, 2016, syn. n.

##### Type species.

*Metopomystrum pehlkei* Günther, 1939 by original designation.

##### Composition and distribution.

Five species, all in northern part of South America (Brazil, Colombia), namely (1) *M. amazoniensis* (Colombia: Amazonas), (2) *M. apterum* (Brazil: Bahia), (3) *M. lilianae* (Colombia: Santander), (4) *M. muriciense* Silva & Skejo, sp. n. (Brazil: Alagoas), and (5) *M. pehlkei* (Colombia: Tolima).

##### Revised generic description.

*Head*. Frontal costa bifurcation situated above the paired lateral ocelli, between the compound eyes, so the frontal costa is long and evident before bifurcation, scutellum narrower than scapus, antennal grooves situated at the level of the lower margins of the compound eyes, paired lateral ocelli situated between the compound eyes, head and eyes exserted above pronotum, eyes protruding, so the vertex is not visible in lateral view, antennae 15-segmented, filiform, with cylindrical segments and smooth margins, fastigium narrower than the compound eyes, lateral carinae of the vertex continuous, not elevated, median carina of the vertex very weak, almost absent, anterior margin of the vertex rounded or acute, fastigium of the vertex projecting forwards and forming a horn longer than combined length of a compound eye and frontal costa above its bifurcation, dorsum of the horn with deep depression formed of joined fossulae, frontal ridge in lateral view with weak excision between the compound eyes.

*Pronotum.* slender in appearance, anterior margin truncated, prozonal carinae present, parallel or slightly diverging, extralateral carinae indistinct, median carina continuous from the anterior margin to the posterior apex, pronotal projections lacking, humeral angle wide, obliquely rounded, interhumeral carinae indistinct, interscapular area in winged species narrow with parallel margins, in wingless species as wide as mid femur, lateral area wider in wingless species, humero-apical, humeral and lateral carinae continuous, not armed or tuberculated, paranota triangular, lateral lobes of the pronotum directed slightly sidewards, with rounded apex lacking ventrolateral spine, dorsum of pronotum between the carinae smooth, pronotal apex truncated or acute.

*Legs.* Fore and mid femora not significantly compressed, fore femora with straight to slightly undulated dorsal and ventral margins, not armed with teeth or spines, mid femora not compressed, carinated above, with straight to slightly undulated dorsal and more undulated ventral margins, hind femora with smooth dorsal and ventral margins, dorsal margin with genicular and antigenicular teeth in hind knee, transverse ridges in the external surface well visible, not armed with lappets or spines, hind tibiae and tarsi not flattened or widened.

##### Revised generic diagnosis.

From other genera previously assigned to Cleostratini or Miriatrini, and from all the South American Metrodorinae genera (*Allotettix* Hancock, 1899; *Amorphopus* Serville, 1838; *Chiriquia* Morse, 1900; *Cota* Bolívar, 1887; *Cotys* Bolívar, 1887; *Crimisus* Bolívar, 1887; *Eomorphopus* Hancock, 1900; *Hancockiella* Cadena-Castañeda & Cardona, 2015; *Metrodora* Bolívar, 1887; *Miriatra* Bolívar, 1906; *Otumba* Morse, 1900; *Platythorus* Morse, 1900; *Plesiotettix* Hancock, 1907; *Scabrotettix* Hancock, 1907 and *Trigonofemora* Hancock, 1906). The genus *Metopomystrum* can be separated by the following set of characters: head exserted above the pronotum, frontal costa long above the bifurcation, antennal grooves situated at the level of the lower margins of the compound eyes, antennae 15-segmented, filiform, paired lateral ocelli situated between the compound eyes, median carina of the vertex weak, indistinct, not projecting forwards and not compressed, fastigium of the vertex with deep depression, lateral carinae of the vertex continuous, pronotum flat, without projections and protuberances, lateral pronotal lobes directed sidewards, having rounded apices, femora without teeth or lappets, fore and mid femora not compressed and flattened.

### New species description

#### Metopomystrum muriciense

Taxon classificationAnimaliaOrthopteraTetrigidae

Silva & Skejo sp. n.

http://zoobank.org/2415AEB8-097F-48C9-8064-7311E629BDCF

Figures 1 , 2 , 3

##### Specimen.

Holotype 1♂, in alcohol. Original label: “Brasil, Alagoas, Murici, Estação Serra do Ouro (9°14.54'S, 35°50.2'W), 21–23/i/2013. C. Sperber e equipe leg.” “[licença Sisbio n° 37717]” (MNRJ). Conservation status: left antenna broken.

##### Type locality.

Brazil: Alagoas state, municipality of Murici, Estação Serra do Ouro, the holotype was caught in forest leaf litter [coordinates 9°14.54'S, 35°50.2'W].

##### Etymology.

The species name refers to the municipality of Murici.

##### Diagnosis.

This species can be distinguished from other species of *Metopomystrum* by the following set of characters: (i) long and acute fastigium, directed forwards, slightly upwards in its apex, (ii) forehead, genae and posthumeral spots in the pronotum yellowish, (iii) tegmina, alae and tegminal sinus absent, (iv) lateral lobes of the pronotum directed slightly sidewards, with rounded apex and yellowish band, (v) sternomentum necklace-shaped, brown with yellowish spots.

##### Comparative notes.

The species can easily be separated from winged Colombian species (*M. lilianae* and *M. pehlkei*) by the horn morphology, fastigium having a rounded anterior margin in those species, while it is acute in *M. muriciense* Silva & Skejo, sp. n., and it is directed upwards in those species, while forwards in *M. muriciense* Silva & Skejo, sp. n. *Metopomystrum lilianae* and *M. pehlkei* are winged species, with visible tegmina, narrower than the maximum width of the mid femora, and tegminal sinus, hence narrow infrascapular area which is wide and evident in apterous *M. muriciense* Silva & Skejo, sp. n. From the apterous Colombian species *M. amazoniensis*, the new species can be separated by the straight horn, not directed upwards as in *M. amazoniensis*. However, the two species only share morphology of the anterior margin, laterally vertex being narrower in *M. muriciense* Silva & Skejo, sp. n. than in *M. amazoniensis*. The occipital area in *M. muriciense* Silva & Skejo, sp. n. is much longer than in Colombian species of the genus. The species is morphologically most similar to *M. apterum*, also from Brazil, with which it shares numerous morphological features, differing however in horn (projection of the fastigium of the vertex) direction-forwards and slightly upwards in *M. muriciense* Silva & Skejo, sp. n., while slightly downwards in *M. apterum*. In *M. muriciense* Silva & Skejo, sp. n. the vertex is narrower and more acute than in *M. apterum*.

##### Holotype description.

**Figure 2.**
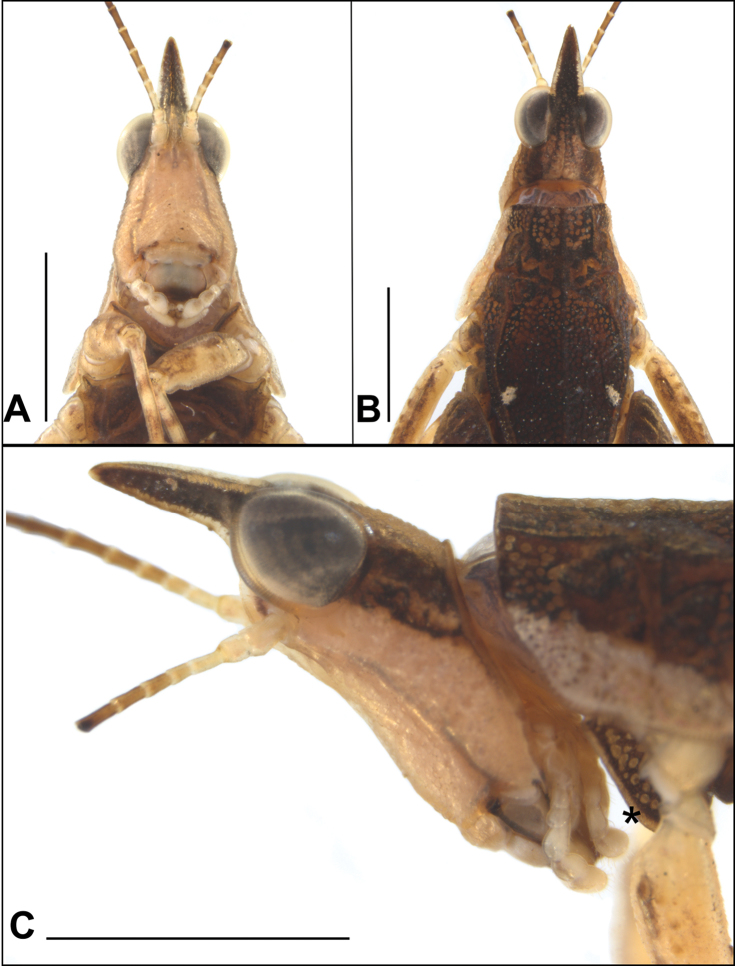
*Metopomystrum muriciense* Silva & Skejo, sp. n.: **A** Male holotype, head and portion of sternum, frontal view **B** head and portion of pronotum, dorsal view **C** head and portion of pronotum, lateral view (* sternomentum). Scale bars: 2.0 mm.

**Figure 3.**
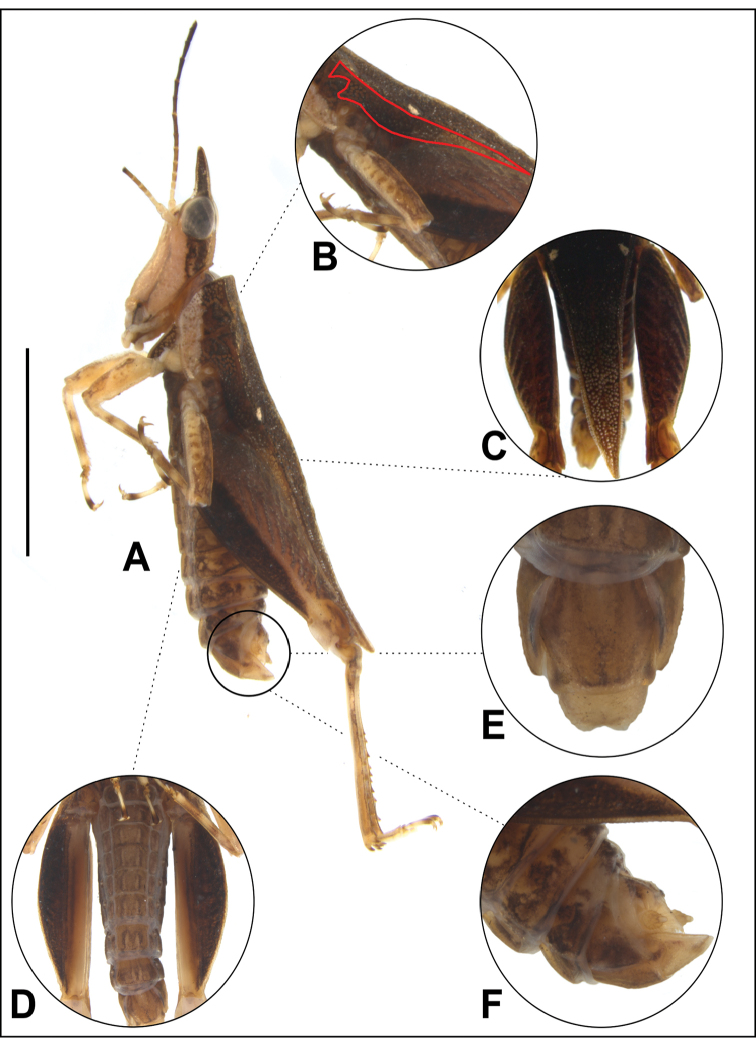
*Metopomystrum muriciense* Silva & Skejo, sp. n.: **A** Male holotype, habitus, lateral view **B** pronotum, lateral view, with infrascapular area marked in red lines **C** distal portion of pronotum with yellowish posthumeral spots and pronotum tip with serrate edge and many yellowish spots, dorsal view **D** abdomen and hind femur, ventral view **E** subgenital plate, dorsal view **F** terminalia, lateral view. Scale bar: 5.0 mm.

***Head.*** In lateral view (Figs 2C, 3A): occipital area elongated and granulated, head and compound eye insert exerted above the pronotal disc; vertex is not visible; fastigium of vertex and frontal carina forming long triangular horn with acute apex that is as long as the compound eye measured from its most frontal point to the apex; frontal carina not projecting; antennal groove situated slightly below the compound eye, almost at the level of its lower margin; palpi compressed, segments enlarging towards apex. In frontal view (Fig. 2A): frontal costa above the bifurcation long; frontal costa bifurcation placed between the compound eyes; scutellum very narrow; scapus two times as wide as scutellum; antennae filiform with 15 antennomeres, segments elongated, cylindrical with smooth margins, paired lateral ocelli situated between the compound eyes, slightly below the frontal costa bifurcation; median ocellus placed in the end of facial carinae, in the place where frontal costa shallowly continues towards the clypeal triangle, of which carinae are also weak and not prominent, in the widest part; head slightly wider than the width between the lateral margins of the compound eyes. In dorsal view (Fig. 2B): vertex between the eyes as wide as a compound eye’s horizontal diameter; fastigium of the vertex triangular, with acute apex; median carina of the vertex inconspicuous; fastigium lowest in the part of median carina, forming depression; occipital area 2× shorter than a compound eye’s vertical diameter and granulated. *Coloration* (Figs 2A–C, 3A): fastigium of the vertex and vertex between the compound eyes dark brown; lateral area between frontal costa and lateral carinae of the vertex dark brown as well; median part of occipital area yellowish, with dark ornamentation; behind the eyes dark brown band with weaker pale brown and yellowish band inside; frons, clypeal triangle and gena pale yellowish; palpi pale; first four antennomeres almost white.

***Pronotum.*** Brachypronotal (because of lack of wings it can be also regarded nanopronotal, organs of flight not visible), reaching abdominal apex. In lateral view (Figs 2C, 3A–B): median carina slightly undulated; prozonal carina visible, short, extralateral carina inconspicuous, visible as a few fine tubercles; ventral sinus absent, covered with infrascapular area; sulci between prozonal and humero-apical carina deep; paranota smooth, with few pits and tubercles, triangularly shaped (in typical way for non-Batrachideinae tetrigids); lateral lobe directed sidewards and truncated in apex; humero-apical carina connected to external lateral carina, which is parallel to median carina; infrascapular area wide, almost as wide as mid femur, with numerous shallow depressions (places where the chitinous layer is thinner, well visible by changing light under stereomicroscope); width of the infrascapular area decreasing towards apex; infrascapular area connected to narrow lateral area; internal lateral carina parallel to external lateral carina; apex of the pronotum directed upwards. In frontal view (Fig. 2A): shoulders not projected, lateral pronotal lobes directed downwards and sidewards. In dorsal view (Figs 2B, 3C): dorsum rich in fine granules, tubercles and small pits (shallow depressions in chitinous layer, look like holes, light permeable); anterior margin of the pronotum truncated, slightly inverted, prozonal carinae short, visible; angle between humero-apical and external lateral carinae obtusely rounded; median carina not strongly elevated, continuous; interhumeral carina indistinct; small posthumeral spots present; pronotum suddenly narrowed in the apex, its apex acute; lateral lobes with weak triangular ventrolateral projection with bluntly truncated apex, directed outwards and backwards. *Coloration*: dorsum brown, holes and granules being pale when enlightened; distal part of the disc, around apex, with numerous pale spots; ventral third of paranota with pale yellowish band; two posthumeral yellowish spots present, left rounded, while right triangular, smaller than observed in other Tetrigidae species.

***Wings.*** Flightless species, tegmina and wing not visible, reduced and covered by infrascapular area of pronotum, not functional. (Note: to check if wings are present pronotum needs to be broken. We did not want to break the pronotum since there is only one specimen, the holotype). *Sternomentum* (Fig. 2C) [= modified prothoracic sternum]. Sternomentum collar-like, necklace shaped, well visible in lateral and ventral view, brown in color, ornamented with numerous yellowish spots of the same shape as pits on pronotal surface.

***Legs*** (Figs 2A–B, 3A–D). *Fore legs*: fore femur approximately rounded in cross-section; dorsal margin carinated, dorsal carina made of fine tubercles, slightly undulated; ventral carina made of fine tubercles too, almost straight; fore femur widened in medial part, narrowed towards proximal and distal connection; distal part of the dorsal margin bearing a blunt tooth; fore tibia robust, slightly longer than fore femur; distal third of the inner margin equipped with four pairs of spines; proximal tarsal segment short, with three rounded pulvilli on its ventral surface; distal tarsal segment longer than the proximal, at its apex with two claws. *Mid legs*: femur with distinctly carinated dorsal and ventral margins; dorsal and ventral carinae of the mid femur visible in lateral view, not very prominent; dorsal and ventral margins finely tuberculated, slightly undulated to almost straight; mid tibia slightly shorter than the mid femur, robust, with small spines on its inner margin; proximal tarsal segment short, with angular pulvilli on ventral surface; distal segment four times as long as proximal, at its apex with claws. *Hind legs*: dorsal and ventral margins of hind femora finely granulated, without lappets or projections; genicular and antigenicular teeth small; clearly visible hind femur elongated and reaching slightly below the pronotal apex when extended; external surface finely granulate, with seven transverse ridges in its external surface (or more if short are counted towards the knee); dorso-external and ventro-external carinae of the hind femur without tubercles or projections, smooth, dorsal margin in dorsal view straight; ventral margin and inner ventral margin of the hind femora straight; tibial sulcus (= the depression on the ventral side of the hind femora in which the tibiae are put when the grasshopper is walking) half as wide as hind femora in ventral view; hind tibiae robust, shorter than hind femora with dorsal surface concave, ventral convex, dorsal surface with two rows of spines from its middle to the connection tarsus; inner margin with nine visible spines, outer with seven, on connection of tibia and tarsus there are 6 spines, three on each side; proximal segment of the hind tarsus with acute pulvilli; mid segment short; distal segment as long as proximal, having two claws in its apex. *Coloration*: fore femur yellowish, with small amount of darker areas in its dorsal part; fore tibia of the same colour as fore femur, with two dark rings, the distal one being broader and darker; proximal segment of fore tarsus dark; one half of the distal segment pale, the other part dark; claws yellowish with dark apex. Mid femur yellowish, with dorsal and distal part being dark and having four dark patches in external surface; mid tibia yellowish, with two dark rings, the distal one being broader and darker; proximal segment of the mid tarsus pale brown; distal segment with two thirds yellowish, apical third dark, claws yellowish with dark apex; ventral external area of the hind femur very dark; median external area lighter brown; dorsal area somewhat darker than median, but lighter than ventral; hind knee yellowish; hind tibia yellowish with brownish patches, not forming dark rings; proximal and mid segment of hind tarsi yellowish, apical part of the distal segment darker, rest of it yellowish; claws light with dark apex.

***Abdomen*** (Figs 3A, 3D–F). Subgenital plate bilobate, with deep triangular incision; each lobe triangular with blunt apex; in lateral view subgenital plate with apex directed upwards; cerci short, robust, their basal part swollen and hirsute. *Coloration*: visible parts of dorsal sternites dark brown, similar to pronotum; upper lateral parts of the sternites light yellowish, from the middle to the connection with sternites ornamented with dark brown and yellowish patches; epiproct and cerci yellowish; subgenital plate light brown; sternites light brown and each of them ornamented with a pair of thin pale lines, having darker margins outside.

##### Female.

Unknown.

##### Measuremens (all in mm).

Body length from the tip of the fastigium projection to the end of the abdomen 10.8; fastigium length 1.03; vertical eye diameter 0.96; horizontal eye diameter 0.67; vertex width 0.41; pronotum length 8.12; pronotum lateral lobes maximal width 1.86; infrascapular area length 5.54; fore femur length 1.61; fore femur width 0.62; fore tibia length 1.63; mid femur length 1.99; mid femur width 0.59; mid tibia length 1.48; hind femur length 5.11; hind femur maximal width 1.67; hind tibia length 4.08; proximal hind tarsal segment length 0.51; mid hind tarsal segment length 0.04; distal hind tarsal segment length 0.50.

### Key to the species of *Metopomystrum*

**Table:** 

1	Fastigium of the vertex and frontal costa forming horn with rounded apex in dorsal view, in frontal and lateral view horn projecting above the eyes for more than one half of a compound eye height. Tegmina and wings visible and surpassing abdominal apex. [Colombia]	**2**
–	Fastigium of the vertex and frontal costa forming horn with acute apex in dorsal view, in frontal and lateral view horn projecting above the eyes for less than one half of a compound eye height. Tegmina and wings not visible, infrascapular area visible in their place.	**3**
2	Horn directed forwards and upwards at about 30^o^ in relation to the vertex between the eyes and pronotal disc. Tegmina and pronotum unicoloured [Colombia: Tolima]	***Metopomystrum pehlkei* Günther, 1939**
–	Horn directed strongly upwards at about 45º in relation to the vertex between the eyes and pronotal discus. Ventral margin of tegmina and dorsal margin of pronotum yellowish. [Colombia: Santander]	***Metopomystrum lilianae* Cadena-Castañeda & Cardona, 2015**
3	Horn directed upwards, elevated in relation to pronotal disc for about one third of a compound eye height, in lateral view horn wide and with rounded apex, scutellum two times narrower than scapus, eyes in lateral view triangular. [Colombia: Amazonas]	***Metopomystrum amazoniensis* Cadena-Castañeda & Cardona, 2015, comb. resurr.**
–	Horn directed almost completely forwards, only slightly elevated above a compound eye, in lateral view horn thin and with triangular apex, scutellum three times narrower than scapus, eyes in lateral view rounded. [Brazil]	**4**
4	Horn directed forwards, slightly upwards, vertex narrow and acute in dorsal and lateral view	***Metopomystrum muriciense* Silva & Skejo, sp. n.**
–	Horn directed forwards, slightly downwards in its apex, vertex wider and more rounded in dorsal and lateral view	***Metopomystrum apterum* Günther, 1939 comb. resurr.**

## Discussion

### Taxonomic and biogeographic considerations on *Metopomystrum* species

Storozhenko’s (2016) division of the genus into two genera is not advisable, because it is based mostly on presence or absence of tegmina and wings, so *Apteromystrum* syn. n. is regarded synonymous with *Metopomystrum* (*Metopomystrum apterum* comb. resurr. and *Metopomystrum amazoniensis* comb. resurr.). Holotype of *Miriatra brevifastigiata* comb. n. is likely a nymph of *Miriatra* close to *M. producta* (see Results-Taxonomic comment on *Metopomystrum brevifastigiata*) .

Günther (1939) stated that *Metopomystrum* was similar to *Miriatra* Bolivar, 1906. We cannot confirm this statement since *Metopomystrum* is very different from *Miriatra* in vertex, pronotum and leg morphology, of slender appearance with weak elevations of pronotal carinae, while *Miriatra* has robust, *Chiriquia*-like appearance, with strong and tuberculated carinae and strong lateral lobes of the pronotum. Frontal projection is wide in *Metopomystrum*, much more similar in form to that in *Procytettix* Bolívar, 1912; *Rhopalotettix* Hancock, 1910 and *Rhynchotettix* Hancock, 1907. *Miriatra* Bolívar, 1906 is unique in having highly laterally compressed median carina of the vertex that is connected to frontal carina, together forming a horn. In pronotal and leg morphology *Miriatra* is similar to members of *Metrodora* Bolívar, 1887, *Cota* Bolívar, 1887, *Chiriquia* Morse, 1900 and *Otumba* Morse, 1900.

Within Miriatrini and Cleostratini, *Metopomystrum* is the only genus comprising winged and wingless species. The problem of division of genera by the presence or lack of wings is widespread in Tetrigoidea taxonomy (e.g., Bolívar 1887, Storozhenko 2016; see *Procytettix* in Bolívar 1912 and Rehn 1937). The species with short pronotum exhibit stronger carination, undulation, and tuberculation of its surface in comparison to species with longer pronotum (e.g., Rehn 1937, Tumbrinck 2014).

*Metopomystrum muriciense* Silva & Skejo, sp. n. is the only known species of the genus that has small posthumeral spots. *Metopomystrum* species have very diverse coloration and probably colour varies within a species. A lot of Tetrigidae species are known to have huge colour pattern variation within and among populations (e.g., Nabours 1929). Variation is a result of numerous factors, including genetics, epigenetics (Karlsson et al. 2009), habitat selection and environmental change (Hochkirch et al. 2008, Forsman et al. 2011), mating (Caesar et al. 2007), temperature effects, camouflage and predator avoidance (Ahnesjö and Forsman 2006, Karlsson and Forsman 2010, Tsurui et al. 2010).

Information on habitat and distributions are lacking for the majority of species of the family Tetrigidae. *Metopomystrum* is not exception. This genus was erected by Günther (1939), who described two species (*M. pehlkei* from Colombia and *M. apterum* comb. resurr. from Bahia). After almost eight decades without records Cadena-Castañeda and Cardona 2015 described three new species for Colombia, of which two are currently assigned to *Metopomystrum* (*M. lilianae* and *M. amazoniensis* comb. resurr.). The genus is not well documented and remains poorly known, all the species being described from only a few available individuals, and the variability remaining completely undocumented. Here we reviewed critically diagnostic characters that unite the reported species and specimens into one taxonomic unit. Even with the new species described herein from Alagoas, knowledge on *Metopomystrum* remains scarce.

The genus has a large gap in the known distribution between Colombia and Brazil (Fig. 1). Serious studies are needed to assess the complete distribution of these tropical rainforest species, to study them and to protect them because pygmy unicorns exhibit rare and interesting morphological features not seen in other tetrigids. We are sure that more species of this genus exist, but they could not be documented yet due to limited zoological expeditions focusing on pygmy grasshopper collection in their distribution areas. On the other hand, many tetrigids species have been described from the gap area, by several different authors from several different collections (e.g., Serville 1838, Walker 1871, Bolívar 1887, Hancock 1907, Bruner 1910), from northern Brazil (Pará): *Amorphopus notabilis* Serville, 1838; *Metrodora gibbinotus* (Bruner, 1910); *Metrodora uniformis* (Bruner, 1910); *Otumba basalis* Bruner, 1910 and *Paurotarsus ruficornis* (Walker, 1871). From Guyana: *Eomorphopus granulatus* Hancock, 1907; *Otumba lobata* Hancock, 1907 and from Surinam *Otumba concinna* (Bolívar, 1887). This can imply that *Metopomystrum* species occur in not so accessible areas and that they are not among the common species of South America.

### Taxonomic comments on genera previously included in Miriatrini and Cleostratini

The group proposed by Cadena-Castañeda and Cardona (2015) and the group proposed by Storozhenko (2016), unifying Miriatrini and *Cleostratus* into Cleostratini, are not evolutionary units, but artificial groups gathering numerous genera with various morphology of fastigium, head, pronotum, and legs, with the single ‘shared’ character being the presence of a horn. Here we provide a brief overview of the diversity of horn morphology within Tetrigidae in groups that developed a long fastigium, frontal costa, or scutellum independently.

**Africa and islands of the Indian Ocean**: members of *Rhynchotettix* Hancock, 1907 (Madagascar, Metrodorinae) is unique genus in having toothed lateral lobes of the pronotum, directed outwards and forwards. *Pseudomitraria* has the frontal carina with part of the scutellum forming the horn, in a very unusual way.

**Asia and Islands of SE Asia, Wallacea, Papua, and Oceania**: *Rostella* Hancock, 1913 (Asia, Metrodorinae) resembles *Pseudomitraria* and *Metopomystrum* in the way the horn is formed in lateral view. However, *Rostella*, contrary to *Metopomystrum*, has a wide scutellum and is in pronotal morphology related to Metrodorinae and Scelimeninae (not Scelimenini) of Asia, such as *Spadotettix* Hancock, 1910; *Indomiriatra* Tinkham, 1939 and *Eucriotettix* Hebard, 1930. *Spadotettix* (India, SE Asia, Metrodorinae) does not have a produced vertex, its length usually being less than half of the eye height, as in *Tetrix subulata* (Linnaeus, 1758), among others. Morphologically, *Spadotettix* is more similar to *Indomiriatra*, formerly assigned to *Spadotettix*. *Indomiriatra* (India, Metrodorinae) and *Spadotettix* have a more produced vertex and stronger angles of the lateral lobes of the pronotum. Both genera are similar to certain members of *Coptotettix* Bolívar, 1887 (Tetriginae) and *Criotettix* Bolívar, 1887 (Scelimeninae), in broken median carina (discontinuous, with flattened parts) of the pronotum and with species with or without laterally projecting lobes, varying even within populations of one species *Indomiriatra provertex* could be regarded as a large species of *Spadotettix*. *Thyrsus* Bolívar, 1887 (Oceania) shares with *Birmana* Brunner von Wattenwyl, 1893 (Myanmar) and *Clinophaestus* Storozhenko, 2013 (Thailand) (last two Tripetalocerinae, Clinophaestini) interesting morphological characters-nymphs of all these genera have flattened antennal segments, the number of antennal segments is eleven, apical ones are reduced, the preapical, medial and sometimes the basal antennomeres are widened, and their hind-femora are very robust. *Thyrsus* is not related to Miriatrini in any sense, probably neither to *Cleostratus*. Members of the genus *Cleostratus* Stål, 1877 are unique in having the frons projecting as a horn, and the bifurcation of the frontal costa being above the compound eyes. Therefore it is left as single genus in Cleostratini since no other species of Tetrigidae exhibit a similar way in which the frons is projecting. Thus, Cleostratini, even monotypic, represent a more logical taxon than to group it with *Miriatra*.

*Rhopalotettix*, *Pseudomitraria* and *Spadotettix* are good examples that it is possible to find high variability of vertex prolongation among species of the same genus. This character is present in other Tetrigidae groups, like *Clinotettix* Bey-Bienko, 1933 (Tetriginae), whose members have a longer vertex than members of *Spadotettix*. Rehn (1938) concluded from examination of *Pseudomitraria* and *Miriatra* that the elongation of the fastigium represents a parallel specialization. Taxonomic considerations from Storozhenko (2016) should be re-evaluated. The main advantage of Storozhenko’s (2016) monograph on Cleostratini is the amount of drawings, photos and catalogued data on Tetrigidae with a horn, but the main disadvantage is erecting a tribe for such a group and synonymizing Cleostratinae. We leave only *Miriatra* in Miriatrini, while we leave only *Cleostratus* in Cleostratini. Other genera remain without tribal placement within Metrodorinae, because solely based on horn morphology (modified fastigium, fastigium and frontal costa, or frontal costa, or frons with scutellum), genera previously unified within Miriatrini are rather diverse. A comprehensive revision is needed with numerous characters (cladistic approach, where comparison is possible among numerous characters in matrix) to provide well-founded basis for taxonomic acts.

We conclude (1) that Cleostratini and Miriatrini stat. ressur. are not synonymous, because *Cleostratus* and *Miriatra* belong to morphologically and biogeographically different pygmy grasshopper groups, despite both of them having a horn, (2) that a prolonged horn occurs in different evolutionary groups of pygmy grasshoppers, and (3) that a prolonged horn is not an adequate character useful in Tetrigidae suprageneric taxonomy.

## Supplementary Material

XML Treatment for Metopomystrum

XML Treatment for Metopomystrum muriciense
